# Assortativity in cognition

**DOI:** 10.1038/s41598-023-30301-y

**Published:** 2023-02-28

**Authors:** Ennio Bilancini, Leonardo Boncinelli, Eugenio Vicario

**Affiliations:** 1 IMT School for Advanced Studies Lucca, Laboratory for the Analysis of compleX Economic Systems, Piazza S. Francesco 19, 55100 Lucca, Italy; 2 Department of Economics and Business, University of Florence, Via delle Pandette 9, 50127 Florence, Italy

**Keywords:** Evolution, Human behaviour, Social behaviour

## Abstract

In pairwise interactions, where two individuals meet and play a social game with each other, assortativity in cognition means that pairs where both decision-makers use the same cognitive process are more likely to occur than what happens under random matching. In this paper, we show theoretically that assortativity in cognition may arise as a consequence of assortativity in other dimensions. Moreover, we analyze an applied model where we investigate the effects of assortativity in cognition on the emergence of cooperation and on the degree of prosociality of intuition and deliberation, which are the typical cognitive processes postulated by the dual process theory in psychology. In particular, with assortativity in cognition, deliberation is able to shape the intuitive heuristic toward cooperation, increasing the degree of prosociality of intuition, and ultimately promoting the overall cooperation. Our findings rely on agent-based simulations, but analytical results are also obtained in a special case. We conclude with examples involving different payoff matrices of the underlying social games, showing that assortativity in cognition can have non-trivial implications in terms of its societal desirability.

## Introduction

This paper investigates a concept of assortativity that happens at the cognitive level, where we posit the existence of two cognitive modes according to the dual-process theory of cognition. In application to the issue of cooperation, we show that assortativity in cognition can play a relevant role in determining the emerging average cooperation.

Assortativity is a broad concept that can be applied to different contexts. In general, assortativity means that individuals are more likely to be engaged in interactions with people that are similar to them along some dimensions. It is related to homophily: the tendency of individuals to associate and bond with similar others (from Ancient Greek: *homoû* + *philíē*, ‘love of the same’)^1,2^. Assortativity is a widespread phenomenon. A large amount of evidence has been collected showing that individuals often stay and interact with similar others, in some form or another: similarities may refer to belonging to the same cultural group, the same social or ethnic group, or the same religion^3,4^. In network theory, the assortativity coefficient measures the correlation between nodes of similar degree^5^. The effects of assortativity have also been studied extensively, e.g., in genetics^6,7^ or for the evolution of cooperation^8,9^. If we think of agents as divided in groups according to some characteristic or action, an index of assortativity can be formalized as the difference in probability of matching with an individual of a group conditional on belonging to that same group rather than to a different one^10^. Preferences may be used to rationalize different types of assortativity^11–14^.

The dual process theory is a paradigm that has become prominent in cognitive psychology and social psychology in the last thirty years. In the dual process framework, the decision making is described as an interaction between an intuitive cognitive processes and a deliberative one. Although different approaches emerge from the literature^15–17^, some common characteristics of the two processes are well established. The intuitive process, also called *system 1* or *type 1*, is fast, automatic, and unconscious, while the deliberative process, also called *system 2* or *type 2*, is slow, effortful and conscious. In evolutionary terms, the intuitive cognitive process is older than the deliberative one, and it is shared with other animals^18^. The existence of two systems in reasoning and decision making is extended to the domain of learning with associative implicit processes and rule-based explicit processes^19,20^.

Cooperation is a central feature of human behavior that differentiates *Homo sapiens* from the other species^21,22^. When people are cooperative they pay a cost to benefit others. The emergence of cooperation as a persistent phenomenon is a major focus of research across different subjects, such as social sciences^23^ and biology^24^. Indeed, the wide empirical evidence on cooperation is puzzling. For social scientists, it is at variance with the paradigmatic rational self-interested individual that is known as *Homo economicus*, even if other-regarding individuals can have reasons to cooperate^25^. For biologists, competition among individuals is at the basis of natural selection, and this is likely to wipe out cooperators though it is not necessarily the case^26^. In the literature on evolutionary game theory, great attention has been devoted to the mechanisms through which selection can favor the evolution of cooperation^27–31^. Recently, the cognitive basis of cooperative decision-making has also been explored, both experimentally^32–34^ and through theoretical modeling^35,36^.

**Figure 1:**
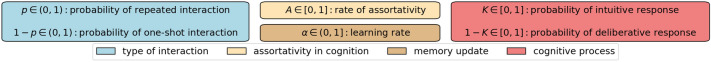
Overview of the model notation, with colors denoting the scope of parameters.

In the following we show that cognition can play an important role for the evolution of cooperation by the channel of assortativity in cognition. By doing so, we exemplify how assortativity in cognition can be incorporated in a fully-fledged model, giving insights on the phenomenon under analysis, namely the emergence of cooperation and the degree of prosociality of intuition and deliberation. To do so, we describe a setting in which agents interact repeatedly in random pairs in two possible types of interaction, the one shot prisoner dilemma, which occurs with probability $1-p$, and the repeated prisoner dilemma, which occurs with probability *p*. As in the previous literature^35^, by repeated prisoner dilemma we mean a stylized representation of an interaction in which there are reciprocal consequences over time: the payoff structure is given by the average payoffs in an infinitely repeated prisoner dilemma in which players can choose between *tit for tat* and *always defect* strategies. Each agent is able to remember the rewards obtained in the past when playing the two different actions, cooperation and defection. This information is stored in the memory of agents. The process of memory update is a form of reinforcement learning: it can be seen as myopic *Q-learning*^37^, i.e., the case in which agents are not able to make any prediction about the future. The process of memory update is characterized by the learning rate $\alpha \in (0,1]$, which represents the weight given to the last reward. We assume that an agent adopts intuition or deliberation depending on the realization of a random variable. In particular, we let $K \in [0,1]$ denote the probability that an agent responds intuitively, so that $1-K$ denotes the probability of deliberation. The cognitive processes adopted by two agents interacting together exhibit assortativity, as measured by parameter $A \in [0,1]$. Indeed, with probability *A* there is a single draw of the random variable, which means that the two agents are forced to use the same cognitive process. With probability $1-A$, there are two independent draws of the random variable, one for each agent, whose cognitive process will be the same or different depending on the realized draws. An overview of the notation is provided in Fig. 1. The details of the model are clarified in “Model” section.

## Results

The results are organized in three parts. In the first one, we provide two theoretical reasons that generate assortativity in cognition, “Sources of assortativity in cognition” section. In the second one we show the simulative results of an applied model on cooperation, “Learning intuitive cooperation through deliberation” section. Finally, in the third one we present the simulative results of two applied models in which small variations from the previous model generate qualitatively different results, “Bivalence of assortativity in cognition on payoffs” section.

### Sources of assortativity in cognition

Assortativity in cognition may arise as a consequence of assortativity on other dimensions, such as the characteristics of the interaction or the characteristics of the interacting agents.

Let *p*(*D*|*D*) be the probability, for a given agent, to interact with a deliberating agent given that the agent is deliberating as well. Following the same notation, *p*(*D*|*I*) is the probability to interact with a deliberating agent given that the agent is deciding intuitively. Let *p*(*I*|*I*) and *p*(*I*|*D*) be defined analogously. There is assortativity in cognition if: $p(D|D)>p(D|I)$, which implies, and is implied by, $p(I|D)<p(I|I)$.

The first source of assortativity in cognition that we examine is state-based assortativity. The characteristics of an interaction (e.g., payoffs, information, complexity of choice) vary across interactions but are often the same, or at least similar, for the individuals in the same interaction. When such characteristics determine the likelihood of deliberation, assortativity in cognition emerges. To fix ideas, consider a case with two states of the world, *A* and *B*, that differ in the likelihood that deliberation and intuition are used by agents. State *A* and state *B* occur with probabilities *p*(*A*) and $p(B)=1-p(A)$, respectively. Agents involved in the same interaction make decisions in the same state. In state *A* an agent decides intuitively with probability $k_A$ while she deliberates with probability $1-k_A$. Analogously, in state *B* an agent decides intuitively with probability $k_B$ while she deliberates with probability $1-k_B$.

In this setting, assortativity in cognition comes out if and only if the likelihood of intuition differs in the two states, i.e., $k_A \ne k_B$ (for the proof see SI Appendix, Subsection 1.1).

The second source of assortativity in cognition that we examine is type-based assortativity. Agents can have heterogeneous characteristics (e.g., skills, abilities, preferences, knowledge) which may determine the likelihood of deliberation. In this case, when the agents participating in the same interaction tend to share the same characteristics, assortativity in cognition emerges. To fix ideas, consider the case where the population is composed by two types of agents, *X* and *Y*, that differ in the likelihood of resorting to deliberation and intuition. The fraction of *X* agents is equal to *p*(*X*) and consequently $p(Y)=1-p(X)$ is the fraction of *Y* agents. Type *X* agents and type *Y* agents decide intuitively with probability $k_X$ and $k_Y$, respectively, while they deliberate with the remaining probability $1-k_X$ and $1-k_Y$. Let *p*(*X*|*X*) and *p*(*X*|*Y*) be the probability of interaction with a type *X* for an agent of type *X* and type *Y*, respectively. There is assortativity in types if $p(X|X)>p(X|Y)$, which implies, and is implied by, $p(Y|X)<p(Y|Y)$.

In this setting, if we assume assortativity in types, then assortativity in cognition comes out if and only if the likelihood of intuition is different for the two types, i.e., $k_X \ne k_Y$ (for the proof see SI Appendix, Subsection 1.2).

### Learning intuitive cooperation through deliberation

**Figure 2:**
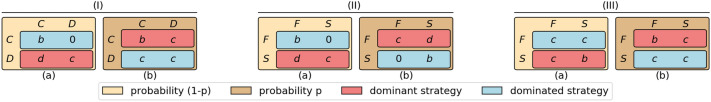
Payoff earned by the row player with $b>c>0$, and $d=b+c$.

Results in this Subsection are based on simulation of the model, where the two interactions are represented by the payoff matrices (I,a) and (I, b) in Fig. 2, with parameters’ values $b=4$ and $c=1$.

**Figure 3:**
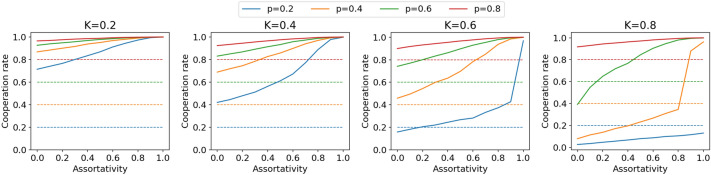
Average cooperation rate varying assortativity in cognition. Each subplot refers to a specific value of *K*. Solid lines represent the average rate of cooperation under intuition, dashed lines represent the average cooperation rate under deliberation, i.e., the value of *p*. Each color refers to a specific value of *p*. Results are based on simulations over 5000 periods, with 500 agents, payoffs $b=4$ and $c=1$, and learning rate $\alpha =0.5$.

A first result is that the average cooperation rate increases monotonically in the level of assortativity in cognition. The result is depicted in Fig. 3 where solid lines represent the average cooperation rate under intuition as the assortativity in cognition varies. Since the cooperation rate under deliberation is constant and equal to *p*, which is depicted with dashed lines in the figure, the result is driven by the increase in cooperation rate under intuition.

A second result points to the existing interaction effect between assortativity in cognition and other parameters in the model. In particular, Fig. 3 suggests that assortativity in cognition can be a substitute for both the likelihood of repeated interactions, i.e., *p*, and the recourse to deliberation, i.e., $1-K$. Indeed, when *p* is quite large there is no room for a significant effect of assortativity in cognition, because repeated interactions are frequent and this, in itself, sustains high rates of intuitive cooperation. Also, when *K* is small every agent frequently deliberates, which implies that often both the agents in an interaction are deliberative, even in the absence of assortativity in cognition.

A third result is an observation that is independent of assortativity in cognition. The average cooperation rate under intuition, for given *p* and *A*, increases as *K* decreases, i.e., the more frequently agents resort to deliberation. Deliberation is able to shape the intuitive heuristic toward cooperation or, in other words, agents learn intuitive cooperation through deliberation.

Finally, a fourth result is about the role of assortativity in cognition in determining whether intuition is more cooperative than deliberation, which is a theme that has been harshly debated in the literature^34,38^. In our model, intuition can be more cooperative than deliberation, or the opposite can happen, and assortativity in cognition plays a role for this. By looking at Fig. 3, we observe that the average cooperation rate is always higher under intuition than under deliberation when *K* is quite small or *p* is quite large. When *K* is large and *p* is small, assortativity in cognition matters: indeed, it is often the case that intuition is still more cooperative than deliberation for high values of assortativity, while deliberation turns out to be more cooperative than intuition when assortativity in cognition is small. In this sense, assortativity in cognition helps intuition to be more cooperative than deliberation, in that it enlarges the region in the set of parameters where this holds.

### Bivalence of assortativity in cognition on payoffs

Drawing from the results in “Learning intuitive cooperation through deliberation” section, one may be tempted to conclude that assortativity in cognition is socially desirable, in that a higher level of assortativity in cognition always leads to a superior societal outcome. In this subsection, we show that this conclusion would be an overstatement: indeed, the effects of assortativity in cognition on welfare, i.e., the sum of payoffs over the whole population, are complicated in general, and hence must be evaluated case by case.

In the previous section we focused on the cooperation rate since the total reward of agents is increasing in it. In the following examples we do not have an action that is always more cooperative than the other action, hence we focus on the average total reward, i.e., the average reward over the whole population along the entire time span.

We replicate the simulations of the previous section changing the types of interaction in which the agents are involved. For simplicity, we consider each of the two interactions in subplot (I) Fig. 2 combined with a variant of it, in which the two actions are permuted, i.e., the actions have inverted payoff consequences in the two types of interaction.

Firstly we consider two one shot prisoner dilemmas, subplot (II) Fig. 2. Under deliberation, agents choose the dominant action, *S* in game (a) (subplot (II), Fig. 2) and *F* in game (b) (subplot (II), Fig. 2). Let *p* be the probability of game (b) (subplot (II), Fig. 2). In this setting, playing the dominated action increases the overall payoff, with the result that miscoordination in behaviors can be beneficial with respect to coordination in the dominant action.

**Figure 4:**
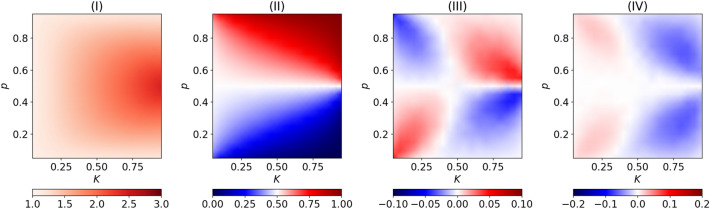
Simulations of the double one shot prisoner dilemma: (I) Average reward with $A=0$; (II) Rate of intuitive play of action F when $A=0$; (III) Variation in the rate of intuitive play of action F passing from $A=0$ to $A=1$; (IV) Variation in the average reward passing from $A=0$ to $A=1$.

Figure 4 shows in (IV) that an increase in assortativity is welfare increasing when *K* is low and welfare decreasing when *K* is high. To grasp the learning effects contributing to this result, we can focus on pairs with one agent intuitive and the other deliberative, given that the main effect of assortativity is to reduce the likelihood of such pairs. Consider $p>0.5$ (everything remains the same when $p<0.5$, with *F* and *S* switched). As *K* increases, i.e., agents are more often intuitive, the probability to choose action *F* gets larger under intuition (Fig. 4, II). Suppose first that the intuitive agent chooses *F*. With probability *p* both agents play *F*, since *F* is dominant and hence surely chosen by the deliberative agent, yielding no substantial effects on learning. With probability $(1-p)$, the deliberative agent chooses *S* because it is dominant, with the result that *S* performs well and *F* performs poorly, which makes *S* more likely to be adopted in the future for both agents. Suppose now that the intuitive agent chooses *S*. Analogously, with probability $(1-p)$ both agents play *S*, with no substantial effect on learning, while with probability *p* the deliberative agent chooses *F* since it is dominant, which triggers a learning effect. Indeed, in the latter case *F* performs well and *S* performs poorly, which makes *F* more likely to be adopted in the future for both agents. Please note that *S* is the welfare enhancing action, when $p>0.5$. To complete the reasoning, we make two observations. A first observation is that the two learning effects described above, one favoring *S* and the other favoring *F*, get weakened when assortativity in cognition increases, due to the reduction in the likelihood that a pair occurs with one agent intuitive and the other deliberative. The second observation is that an increase in *K* raises the likelihood of the learning effect favoring *S* and decreases the likelihood of the learning effect favoring *F*. This is so because a larger *K* makes the intuitive player more often choose *F* (Fig. 4, II), and the intuitive agent has to play *F* for the former effect and *S* for the latter effect. Therefore, an increase in assortativity reduces the likelihood of playing the dominant action when *K* is low and increases it when *K* is high (Fig. 4, III). Since the dominated action is socially optimal, this leads us to conclude that assortativity in cognition is welfare enhancing for low values of *K* and welfare decreasing for high values of *K* (Fig. 4, IV).

Secondly, we consider two repeated prisoner dilemmas, subplot (III) Fig. 2. Under deliberation, agents choose the weakly dominant action, *S* in game (a) (subplots, Fig. 2) and *F* in game (b) (subplots, Fig. 2). Let *p* be the probability of game (b) (subplots, Fig. 2). In this setting average payoffs are maximized when both agents choose the weakly dominant action, while other outcomes pay the same.

**Figure 5:**
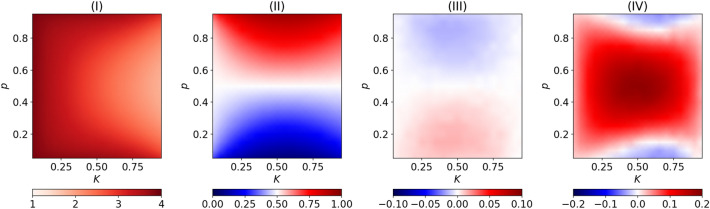
Simulations of the double repeated prisoner dilemma: (I) Average reward with $A=0$; (II) Rate of intuitive play of action F when $A=0$; (III) Variation in the rate of intuitive play of action F passing from $A=0$ to $A=1$; (IV) Variation in the average reward passing from $A=0$ to $A=1$.

Intuitively, greater deliberation, i.e., a lower *K*, is beneficial because it makes agents choose the weakly dominant action (Fig. 5, I). The average payoff also increases for extreme values of *p*, close to either 0 or 1 (again Fig. 5, I), because also intuitive agents choose the weakly dominant action most of the time (Fig. 5, II). As already pointed out in the previous subsection, assortativity in cognition decreases the probability of interaction between an intuitive agent and a deliberative one, thus increasing the probability of interaction between two intuitive agents and between two deliberative agents. On the one hand, an increase of assortativity yields a direct effect on payoffs in that the increased likelihood of two deliberative agents interacting together allows an easier coordination on the weakly dominant action. On the other hand, there are other effects triggered by learning. To grasp these learning effects, we focus again on pairs with an intuitive agent and a deliberative one. Consider $p>0.5$ (everything remains the same when $p<0.5$, with *F* and *S* switched). The most likely occurrence here is that agents play game (b) (Fig. 5), which happens with probability *p*, and that the intuitive agent plays action *F* (Fig. 5, II). Since the deliberative agent surely chooses *F* as well, they obtain the highest payoff *b*, which increases the likelihood of playing action *F* in the future. The least likely occurrence is that agents play game (a) (Fig. 5), which happens with probability $1-p$, and that the intuitive agent plays action *S* (Fig. 5, II). Since the deliberative agent surely chooses *S* as well, they obtain the highest payoff *b*, which increases the likelihood of playing action *S* in the future. Since action *F* is more often the weakly dominant action, given $p>0.5$, the former effect is stronger than the latter. To complete the picture, there are other two cases in which the intuitive agent plays the dominated action, this yielding no substantial effect on learning because both agents earn a payoff equal to *c*, even if for different actions. Overall, an increase in assortativity in cognition leads to a decrease in the rate at which intuitive agents play the action that is more often dominant (Fig. 5, III). In turn, this has a negative impact on average payoffs, and this impact is greater for extreme values of *p*, close to either 0 or 1 (Fig. 5, III). It turns out that, for extreme values of *p*, close to either 0 or 1, this negative indirect effect through learning more than offsets the positive direct effect on payoffs, resulting in the blue areas in Fig. 5, IV.

## Discussion

Assortativity is a phenomenon characterizing social interactions in many contexts and along different dimensions. Our work explores a new dimension of assortativity, occurring at the cognitive level: the agents involved in the same interaction often exhibit similar degrees of cognitive effort. To the best of our knowledge, assortativity in cognition has not been considered and analyzed by the literature so far. In some cases, it is involved or even implied, but the focus was never on it. For instance, priming has been shown to affect the activation of cognitive processes^39^, hence interacting partners who are exposed to the same priming are more likely to rely on the same cognitive process. Recently, the connection between cognitive reflection and behavior in social media platforms was investigated^40^, identifying the existence of cognitive echo chambers in which users with similar cognitive reflection tend to cluster. Also, assortativity in actions often implies assortativity in cognition as a byproduct^35^. Assortativity in cognition along the temporal dimension emerges in evolutionary game theoretic models where cognitive processing and the environment in which agents interact affect each other^41,42^.

When assortativity in cognition emerges through assortativity in types, it also comes with assortativity in behavior^35^, at least if types are defined including actions. When this is the case, it is impossible to disentangle the effect of assortativity in cognition from the effect of assortativity in behavior. Our result in “Learning intuitive cooperation through deliberation” section suggests that assortativity in cognition is able to promote cooperation *per sé*, also in the absence of other forms of assortativity. This result is robust to changes, when we consider different entries in the payoff matrix (SI Appendix, Subsection 3.1) and different learning rates (SI Appendix, Subsection 3.2). The findings in “Learning intuitive cooperation through deliberation” section are based on simulations over 5000 periods, with 500 agents, payoffs $b=4$ and $c=1$, and learning rate $\alpha =0.5$. A greater value of *b* makes cooperation more profitable in the repeated interaction while, in the one shot interaction, it has the same effect on cooperation and defection. Thus, greater values of *b* promote intuitive cooperation. In the SI Appendix (Subsection 3.3) we consider a variant in which deliberative decisions are based on myopic Q-learning with finer information, distinguishing between past performance of cooperation and defection under deliberation in the two types of interaction. We show that qualitatively similar results hold in that case as well for $\alpha < 1$: after relatively few periods agents learn to play the dominant strategy under deliberation, thus yielding substantially equivalent simulations once learning has occurred.

We stress that *K* is homogeneous and exogenous in our model. This is so because our aim is not to study the evolution of dual process reasoning, rather we want to focus on the effects of assortativity in cognition given dual process reasoning, for which the literature has already provided evolutionary arguments^35,43,44^. The exogeneity of *K* is also the reason why, differently from previous contributions in the literature^35^, we do not consider any cost of deliberation, in that deliberation is not modeled as a choice. Quite interestingly, we find that in our model the value of *K* that maximizes the average payoff is often strictly in between 0 and 1 (SI Appendix, Section 4).

In conclusion, assortativity in cognition rests on sound theoretical reasons and yields relevant consequences, in that it allows internalizing the external effects of one’s own cognition: the partner exerts a similar cognitive effort and hence behaves in a similar way. The evolution of behaviors is significantly affected by assortativity in cognition, with consequences on overall welfare that should be carefully evaluated case-by-case.

## Model

Time is discrete and agents are randomly matched pairwise in each period to play one of two possible types of interaction. We consider three applications of the model that are based on different pairs of interaction, as represented in the three subplots of Fig. 2. In the following we describe the functioning of the model when payoffs are those in subplot (I), stressing that the only difference with the other applications is given by the underlying payoff matrices.

The two types of interaction are the one shot prisoner dilemma, game (a), Fig. 2 subplot (I), that occurs with probability $1-p$, and the repeated interaction, game (b), Fig. 2 subplot (I), that occurs with probability *p*. Two actions are available in both interactions, namely cooperation, *C*, and defection, *D*. When the two agents in an interaction play *C*, they both earn *b* irrespectively of the type of interaction. Similarly, when the two agents in an interaction play *D*, they both earn *c* irrespectively of the type of interaction. When the two agents choose different actions, the payoffs depend on the type of interaction: in the one-shot prisoner dilemma, the defecting player earns $d=b+c$ and the cooperating agent earns 0; in the repeated prisoner dilemma, both agents earn *c*. We assume that $b>c>0$, which makes *D* strictly dominant in the one-shot interaction, and *C* weakly dominant in the repeated interaction. This payoff structure is already used in the literature^35^, with the only difference that *c* is added in every cell to avoid negative values.

**Figure 6:**
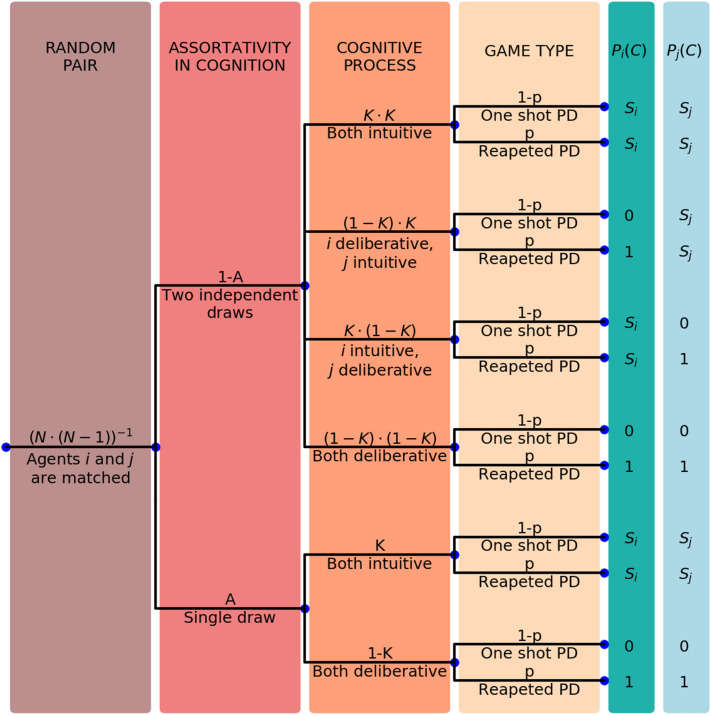
Probabilities are written above branches. For $h \in \{i,j\}$, $P_h(C)$ is the probability of cooperation for agents *h* and $S_h$ is the probability that ${\overline{R}}_{h,C}^t > {\overline{R}}_{h,D}^t$ plus half the probability that ${\overline{R}}_{h,C}^t = {\overline{R}}_{h,D}^t$.

Agents are characterized by their memory, in which are stored information about the past rewards obtained choosing the two different actions. In each period, every agent update the information about the past rewards obtained with the played in that period, keeping unchanged the information about the past rewards obtained with the other action. Indeed the memory of a generic agent *i* at time *t*, $m_i^t$, is made of two elements, the information about the past rewards obtained in the previous periods when playing cooperation, ${\overline{R}}_{i,C}^t$, and the information about the past rewards obtained in the previous periods when playing defection, ${\overline{R}}_{i,D}^t$:

$$\begin{aligned} m_i^t=\{{\overline{R}}_{i,C}^t, {\overline{R}}_{i,D}^t \} \end{aligned}$$

In particular, if agent *i* plays cooperation at time *t*, then the agent’s memory is updated in the following way:

$$\begin{aligned} {\overline{R}}_{i,C}^t & = (1-\alpha ) {\overline{R}}_{i,C}^{t-1}+\alpha R_i^t\\ {\overline{R}}_{i,D}^t &= {\overline{R}}_{i,D}^{t-1} \end{aligned}$$

with $\alpha \in \left( 0,1 \right]$ measuring the learning rate and $R_i^t$ being the reward obtained in the last period. Analogously, if agent *i* plays defection at time *t*, then the agent’s memory is updated in the following way:

$$\begin{aligned} {\overline{R}}_{i,C}^t & = {\overline{R}}_{i,C}^{t-1} \\ {\overline{R}}_{i,D}^t & = (1-\alpha ) {\overline{R}}_{i,D}^{t-1}+\alpha R_i^t \end{aligned}$$

We note that, when the learning rate $\alpha$ is equal to one, only the last reward obtained for each action matters.

The decision process used by agents relies on either intuition or deliberation, with the latter following a more consequentialist rule (based on best reply) than the former (based on reinforcement learning).

- Under intuition the agent is not able to recognize the type of occurring interaction. The intuitive decision is based on the information saved in memory. The action with the highest past reward is chosen: when $\small {\overline{R}}_{i,C}^t> {\overline{R}}_{i,D}^t$ cooperation is chosen, conversely defection is chosen when $\small {\overline{R}}_{i,C}^t< {\overline{R}}_{i,D}^t$. In case of a tie, i.e., when $\small {\overline{R}}_{i,C}^t = {\overline{R}}_{i,D}^t$, each action is chosen with one-half probability.
- Under deliberation the agent is able to recognize the type of occurring interaction. The deliberative decision is driven by best-response. Defection is chosen in the one-shot prisoner dilemma because strictly dominant, while cooperation is chosen in the repeated prisoner dilemma, because weakly dominant.

Assortativity in cognition is measured with the parameter $A \in (0,1]$. Given each pair, with probability *A* the two agents are forced to use the same cognitive process, while with probability $1-A$ the cognitive processes of the two agents are independent. Each agent has probability $K \in [0,1]$ to rely on intuition and probability $1-K$ to rely on deliberation. The possible occurrences, with the associated probabilities, are represented in Fig. 6.

### Markov process

When the learning rate $\alpha$ is equal to one, the behavior of one agent *i*, given the behavior of all the other agents, in the model can be described through a *discrete-time Markov process* *P*, defined on a finite state space *S* and characterized by a transition matrix *T*. The state space is made by all the feasible memories of agent *i*, i.e., all the pairs $\small \{{\overline{R}}_{i,C}^t, {\overline{R}}_{i,D}^t \}$. The transition matrix describes the probabilities of moving from each state to any other. Transition probabilities depend on the current memory, i.e., the state, the parameters *K* and *p*, and the probability of intuitive cooperation of the rest of the population, denoted by ${\overline{x}}$. A probability distribution $\pi$ defined on *S* is a vector of probabilities such that $\sum _{m} \pi _m=1$, where $m \in S$ denotes a memory and $\pi _m$ the probability that the agent has memory *m*. A probability distribution is said invariant if:

$$\begin{aligned} \pi T = \pi \end{aligned}$$

In words, an invariant distribution remains unchanged in the Markov process as time progresses. Since the Markov process has a unique recurrent class, the invariant distribution exists and is unique. Once obtained the invariant distribution, the probability of cooperation under intuition for agent *i* is the sum of probabilities, in the invariant distribution, of states in which $\small {\overline{R}}_{i,C}^t > {\overline{R}}_{i,D}^t$ plus half of the sum of probabilities of states in which $\small {\overline{R}}_{i,C}^t = {\overline{R}}_{i,D}^t$. Indeed, when $\small {\overline{R}}_{i,C}^t > {\overline{R}}_{i,D}^t$ agents cooperate under intuition while they randomly choose the intuitive response in the cases in which $\small {\overline{R}}_{i,C}^t = {\overline{R}}_{i,D}^t$. We denote with $x_i$ the probability of intuitive cooperation in the invariant distribution for agent *i*. Finally, we introduce the consistency condition: in the long run equilibrium of the model, the cooperation rate of agent *i* is equal to the cooperation rate of the other agents, i.e., ${\overline{x}}=x_i$.

**Figure 7:**
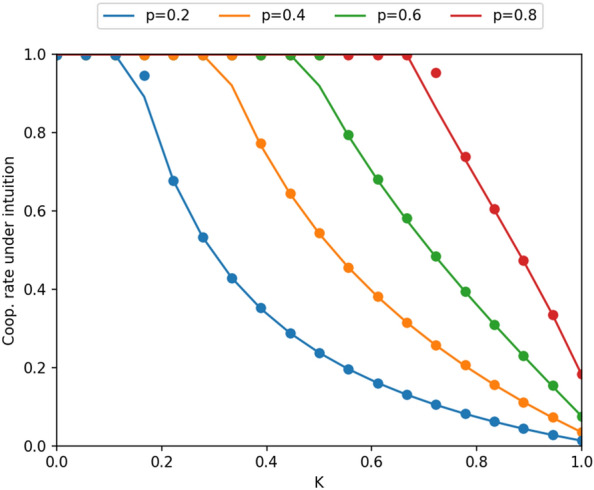
Solid lines are the theoretical frequencies obtained through the long-run Markov chain analysis. Dots are the empirical frequencies obtained through simulations with 500 agents, 5000 time periods, and $A=1$.

In the SI Appendix (Subsection 2.1) we develop the analysis in detail for the simplifying case of full assortativity, i.e., $A=1$.

Figure 7 represents the cooperation rate under intuition, distinguishing between the empirical frequencies obtained through simulations and the theoretical frequencies resulting from the long-run Markov chain analysis. For most values of *p* and *k*, the theoretical analysis overlap with simulations, with only perceptible differences for cooperation rates that are very close to one. See the SI Appendix (Subsection 2.2) for more details on this.

## Supplementary Information

Supplementary Information.

## Data Availability

The datasets generated and analysed during the current study are available from the corresponding author on reasonable request.

## Code availability

*Accession codes*: The code in Python is available at https://github.com/EugenioVicario/Assortativity_in_Cognition.
