# Supplementary material for: Assortativity in cognition
Source: Sci Rep. 2023 Feb 28;13:3412. doi: 10.1038/s41598-023-30301-y (PMC9974973; doi:10.1038/s41598-023-30301-y)
Supplement: Supplementary file 1 — Supplementary Information. [file 41598_2023_30301_MOESM1_ESM.pdf]

Assortativity in cognition

Ennio Bilancini[1], Leonardo Boncinelli[2], and Eugenio Vicario[2]

[1]IMT School for Advanced Studies Lucca, Laboratory for the Analysis of compleX Economic Systems, Piazza S. Francesco 19, Lucca, 55100, Italy

[2]Department of Economics and Business, University of Florence, Via delle Pandette 9, 50127 Firenze, Italy

February 10, 2023

Contents

1 Models of assortativity in cognition **1**
1.1 State-based assortativity 1
1.2 Type-based assortativity 2

2 Theoretical analysis **3**
2.1 Markov chain 3
2.2 Analytical vs. simulative results 6

3 Robustness analysis **7**
3.1 Payoff matrix 7
3.2 Q-Learning 8
3.3 Q-Learning under deliberation 9

4 The optimality of dual process reasoning **11**

1 Models of assortativity in cognition

In the following two subsections we provide simple models where assortativity in cognition arises as a consequence of state-based assortativity (1.1) and type-based assortativity (1.2).

1.1 State-based assortativity

There are two states of the world, A and B, that occur with probabilities $p(A)$ and $p(B) = 1 - p(A)$, respectively. We assume $p(A) \in (0, 1)$. Agents involved together in an interaction are in the same state of the world, i.e., there is full assortativity in the state of the world. Suppose that in state A there is a probability k_A of intuition and a probability $1 - k_A$ of deliberation. Analogously, k_B and $1 - k_B$ are the

probabilities of intuition and deliberation, respectively, in state B. We denote with $p(D|D)$ the probability for an agent, conditional on being deliberative, to interact with an agent who is deliberative as well. Following the same notation, $p(D|I)$ is the probability to interact with a deliberative agent, conditional on being intuitive. Assortativity in cognition occurs when:

$$p(D|D) > p(D|I) \tag{1}$$

From inequality 1, it follows that $p(I|D) < p(I|I)$. Applying Bayes' formula and the definition of conditional probability, inequality 1 can be rewritten as:

$$\frac{p(D \cap D)}{p(D \cap I)} > \frac{p(D)}{p(I)} \tag{2}$$

Let:

$$\begin{aligned} p(D) &= p(A)(1-k_A) + p(B)(1-k_B) \\ p(I) &= p(A)k_A + p(B)k_B \\ p(D \cap D) &= p(A)(1-k_A)^2 + p(B)(1-k_B)^2 \\ p(D \cap I) &= p(A)(1-k_A)k_A + p(B)(1-k_B)k_B \end{aligned}$$

Substituting in inequality 2, the result is:

$$\frac{p(A)(1-k_A)^2 + p(B)(1-k_B)^2}{p(A)(1-k_A)k_A + p(B)(1-k_B)k_B} > \frac{p(A)(1-k_A) + p(B)(1-k_B)}{p(A)k_A + p(B)k_B} \tag{3}$$

Multiplying for the inverse of the right hand side, we obtain:

$$\frac{p(A)^2(1-k_A)^2k_A + p(B)^2(1-k_B)^2k_B + p(A)p(B)\left[(1-k_A)^2k_B + (1-k_B)^2k_A\right]}{p(A)^2(1-k_A)^2k_A + p(B)^2(1-k_B)^2k_B + p(A)p(B)\left[(1-k_A)(1-k_B)(k_A+k_B)\right]} > 1 \tag{4}$$

The numerator and denominator are identical except for the two parts inside square brackets. Then inequality 4 can be rewritten as:

$$(1-k_A)^2k_B + (1-k_B)^2k_A > (1-k_A)(1-k_B)(k_A+k_B) \tag{5}$$

The inequality can be reduced to:

$$(k_A - k_B)^2 > 0 \tag{6}$$

In conclusion, there exists assortativity in cognition, as defined in inequality 1, if and only if $k_A \neq k_B$.

1.2 Type-based assortativity

The population comprises two types of agents, X and Y. The fraction of X agents is equal to $p(X)$ and, consequently, $p(Y) = 1 - p(X)$ is the fraction of Y agents. We assume $p(X) \in (0,1)$. The two types differ in the probability to respond intuitively or deliberately. Assume that type X agents respond intuitively with probability k_X while they deliberate with probability $1 - k_X$. The probability of intuitive and deliberative answers for type Y agents are, respectively, k_Y and $1 - k_Y$. Assume that there exists assortativity in types, i.e., the probability to interact with a type X agent is greater for a type X agent than for a type Y agent:

$$p(X|X) > p(X|Y) \tag{7}$$

From inequality 7, it follows $p(X|Y) < p(Y|Y)$. As in the previous subsection, assortativity in cognition is defined as:

$$p(D|D) > p(D|I) \tag{8}$$

Applying again Bayes' formula and the definition of conditional probability, let::

$$\frac{p(D \cap D)}{p(D \cap I)} > \frac{p(D)}{p(I)} \tag{9}$$

$$\begin{aligned}
p(D) &= p(X)(1-k_X) + p(Y)(1-k_Y) \\
p(I) &= p(X)k_X + p(Y)k_Y \\
p(D \cap D) &= p(X)p(X|X)(1-k_X)^2 + p(X)p(Y|X)(1-k_X)(1-k_Y) + \\
&\quad + p(Y)p(X|Y)(1-k_Y)(1-k_X) + p(Y)p(Y|Y)(1-k_Y)^2 \\
p(D \cap I) &= p(X)p(X|X)(1-k_X)k_X + p(X)p(Y|X)(1-k_Y)K_X + \\
&\quad + p(Y)p(X|Y)(1-k_X)k_Y + p(Y)p(Y|Y)81-k_Y)k_Y
\end{aligned}$$

Proceeding analogously as for state-based assortativity, if $p(X) \neq 0$, $p(Y) \neq 0$ and $k_X \neq k_Y$, then inequality 9 is equivalent to:

$$p(D|D) > p(D|I) \tag{10}$$

In conclusion, if there is assortativity in types and the probability of deliberation is different for the two types, then assortativity in cognition emerges.

2 Theoretical analysis

2.1 Markov chain

When the learning rate α is equal to one, the behavior of one agent i, given the behavior of all the other agents, in the model can be described through a *discrete-time Markov process* P, defined on a finite state space S and characterized by a transition matrix T. The state space is made by all the feasible memories of agent i, i.e., all the pairs $\{\overline{R}_{i,C}^t, \overline{R}_{i,D}^t\}$. The transition matrix describes the probabilities of moving from each state to any other. Transition probabilities depend on the current memory, i.e., the state, the parameters K and p, and the probability of intuitive cooperation of the rest of the population, denoted by $\overline{x}$. A probability distribution π defined on S is a vector of probabilities such that $\sum_m \pi_m = 1$, where $m \in S$ denotes a memory and π_m the probability that the agent has memory m. A probability distribution is said invariant if:

$$\pi T = \pi$$

In words, an invariant distribution remains unchanged in the Markov process as time progresses. Since the Markov process has a unique recurrent class, the invariant distribution exists and is unique. Once obtained the invariant distribution, the probability of cooperation under intuition for agent i is the sum of probabilities, in the invariant distribution, of states in which $\overline{R}_{i,C}^t > \overline{R}_{i,D}^t$ plus half of the sum of probabilities of states in which $\overline{R}_{i,C}^t = \overline{R}_{i,D}^t$. Indeed, when $\overline{R}_{i,C}^t > \overline{R}_{i,D}^t$ agents cooperate under intuition while they randomly choose the intuitive response in the cases in which $\overline{R}_{i,C}^t = \overline{R}_{i,D}^t$. We denote with x_i the probability of intuitive cooperation in the invariant distribution for agent i. Finally, we introduce the consistency condition: in the long run equilibrium of the model, the cooperation rate of agent i is equal to the cooperation rate of the other agents, i.e., $\overline{x} = x_i$.

For the sake of simplicity, we focus on the case of perfect assortativity. Thus the information about past rewards when playing cooperation $\overline{R}^t_{i,C}$ belongs to $\{0, c, b\}$ and analogously $\overline{R}^t_{i,D}$ belongs to $\{c, d\}$, where $d = b + c$. Hence the memory of each agent belongs to the Cartesian product of the column vector $[0, c, b]$ and the row vector $[c, d]$.

To describe the transition probability from state to state, we firstly need to write the probabilities of obtaining a certain reward with cooperation and defection conditioned on the state:

$$\begin{array}{ll} P\left[R_C = r_c | m\right] & \text{with } r_c \in [0, c, b] \text{ and } m \in S \\ P\left[R_D = r_d | m\right] & \text{with } r_d \in [c, d] \text{ and } m \in S \end{array}$$

The probabilities of obtaining a certain reward with cooperation and defection depend on the state on which they are conditioned. The states space can be partitioned in three subsets. In the first one, labeled with S_1, there are the states in which $\overline{R}^t_{i,C} > \overline{R}^t_{i,D}$. In the second group S_2 there are the states in which $\overline{R}^t_{i,C} = \overline{R}^t_{i,D}$, and in the third group S_3 the remaining states in which $\overline{R}^t_{i,C} < \overline{R}^t_{i,D}$. The probabilities of obtaining a certain reward with cooperation and defection depend on the parameters K, p, and $\overline{x}$.

In S_1 the probabilities of different rewards are:

$$\begin{array}{lcl} P\left[R_C = 0 | m \in S_1\right] & = & K(1-p)(1-\overline{x}) \\ P\left[R_C = c | m \in S_1\right] & = & Kp(1-\overline{x}) \\ P\left[R_C = b | m \in S_1\right] & = & (1-K)p + K\overline{x} \\ P\left[R_D = c | m \in S_1\right] & = & (1-K)(1-p) \\ P\left[R_D = d | m \in S_1\right] & = & 0 \end{array}$$

In S_2 the probabilities of different rewards are:

$$\begin{array}{lcl} P\left[R_C = 0 | m \in S_2\right] & = & \frac{1}{2}K(1-p)(1-\overline{x}) \\ P\left[R_C = c | m \in S_2\right] & = & \frac{1}{2}Kp(1-\overline{x}) \\ P\left[R_C = b | m \in S_2\right] & = & (1-K)p + \frac{1}{2}K\overline{x} \\ P\left[R_D = c | m \in S_2\right] & = & (1-K)(1-p) + \frac{1}{2}K(1-\overline{x}) + \frac{1}{2}Kp\overline{x} \\ P\left[R_D = d | m \in S_2\right] & = & \frac{1}{2}K(1-p)\overline{x} \end{array}$$

In S_3 the probabilities of different rewards are:

$$\begin{array}{lcl} P\left[R_C = 0 | m \in S_3\right] & = & 0 \\ P\left[R_C = c | m \in S_3\right] & = & 0 \\ P\left[R_C = b | m \in S_3\right] & = & (1-K)p \\ P\left[R_D = c | m \in S_3\right] & = & (1-K)(1-p) + K(1-\overline{x}) + Kp\overline{x} \\ P\left[R_D = d | m \in S_3\right] & = & K(1-p)\overline{x} \end{array}$$

Starting from the probabilities of obtaining a certain reward with cooperation and defection conditioned on the memory, it is straightforward to build the transition probabilities between different states. To give an example, we focus on the transition probabilities from the state $\{c, c\}$. The probability of transition in one step from $\{c, c\}$ to $\{0, d\}$ or $\{b, d\}$ is equal to zero. Indeed, at least two steps are required to change both the rewards stored in memory. The probability of the transition from $\{c, c\}$ to $\{c, d\}$ is equal to $P\left[R_D = d | \{c, c\}\right]$, while the probabilities of the transitions from $\{c, c\}$ to $\{0, c\}$ and $\{b, c\}$ are, respectively, $P\left[R_C = 0 | \{c, c\}\right]$ and $P\left[R_C = b | \{c, c\}\right]$. Finally, the probability to remain in $\{c, c\}$ is equal to the probability of obtaining

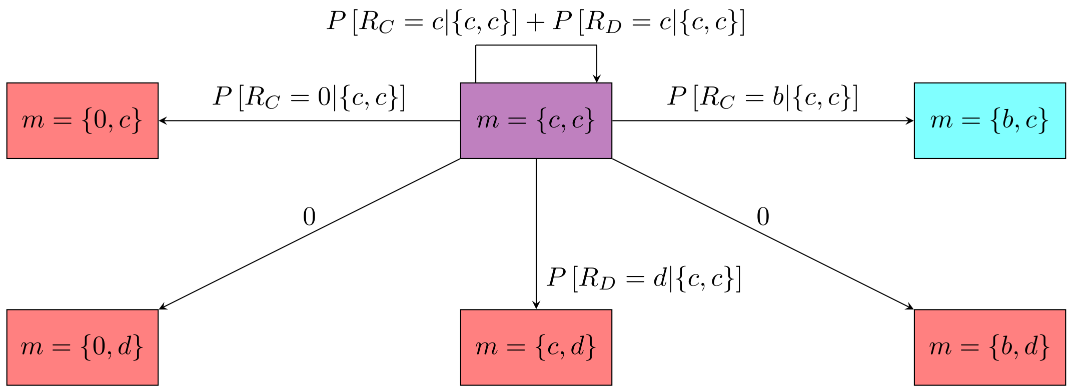

Figure 1: Transition probabilities from state $\{c,c\}$. Colors blue, violet, and red denote states belonging to S_1, S_2, and S_3, respectively.

again c from cooperation plus the probability of obtaining again c from deliberation: $P[R_C = c|\{c,c\}] + P[R_D = c|\{c,c\}]$. See Figure 1 for graphical representation.

In the transition matrix the entry $T_{m_i m_j}$ represents the probability to have a transition from state m_i to state m_j. The summation of all the entries along every row is equal to one.

$$\begin{array}{c} \\ \{0,c\} \\ \{c,c\} \\ \{b,c\} \\ \{0,d\} \\ \{c,d\} \\ \{b,d\} \end{array} \begin{array}{c} \begin{array}{cccccc} \{0,c\} & \{c,c\} & \{b,c\} & \{0,d\} & \{c,d\} & \{b,d\} \end{array} \\ \begin{pmatrix} T_{\{0,c\}\{0,c\}} & 0 & T_{\{0,c\}\{b,c\}} & T_{\{0,c\}\{0,d\}} & 0 & 0 \\ T_{\{c,c\}\{0,c\}} & T_{\{c,c\}\{c,c\}} & T_{\{c,c\}\{b,c\}} & 0 & T_{\{c,c\}\{c,d\}} & 0 \\ T_{\{b,c\}\{0,c\}} & T_{\{b,c\}\{c,c\}} & T_{\{b,c\}\{b,c\}} & 0 & 0 & 0 \\ T_{\{0,d\}\{0,c\}} & 0 & 0 & T_{\{0,d\}\{0,d\}} & 0 & T_{\{0,d\}\{b,d\}} \\ 0 & T_{\{c,d\}\{c,c\}} & 0 & 0 & T_{\{c,d\}\{c,d\}} & T_{\{c,d\}\{b,d\}} \\ 0 & 0 & T_{\{b,d\}\{b,c\}} & 0 & 0 & T_{\{b,d\}\{b,d\}} \end{pmatrix} \end{array}$$

As stated above the Markov chain has a unique recurrent class and thus the invariant distribution exists and is unique:

$$\pi T = \pi$$

The probability of each state in π is a function of the probability of intuitive cooperation $\overline{x}$.

$$\pi = \left[\pi_{\{0,c\}}(\overline{x}), \pi_{\{c,c\}}(\overline{x}), \pi_{\{b,c\}}(\overline{x}), \pi_{\{0,d\}}(\overline{x}), \pi_{\{c,d\}}(\overline{x}), \pi_{\{b,d\}}(\overline{x})\right]$$

The probability of intuitive cooperation is equal to the probability of being in a state belonging to S_1 plus half the probability of being in a state belonging to S_2.

$$x_i = \pi_{\{b,c\}}(\overline{x}) + \frac{1}{2}\pi_{\{c,c\}}(\overline{x}) \tag{11}$$

For the consistency condition we should have:

$$x_i = \overline{x} \tag{12}$$

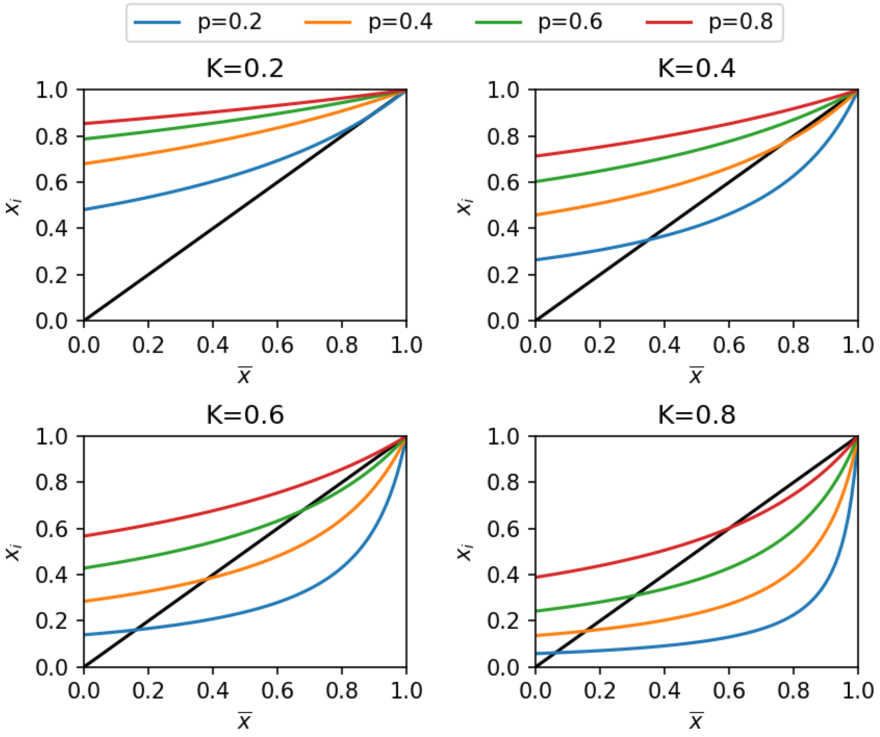

Figure 2: Colored lines represent $x_i(\overline{x})$ for $A = 1$ and different values of p. The intersection with the 45° line identifies the equilibria satisfying the consistency condition $x_i = \overline{x}$. Each subplot refers to a different value of K.

Solving Equation 11, considering the consistency condition in equation 12, we obtain the equilibrium values of x_i and $\overline{x}$, let $x = x_i = \overline{x}$. For all the values of $K \in (0, 1)$ and $p \in (0, 1)$, $x = 1$ is always a solution. When all the agents intuitively cooperate, the reward of cooperation remains higher than the reward of defection whatever the type of interaction and the cognitive mode chosen. When large values of K are associated with low values of p, a second solution emerges in between 0 and 1.

The analysis carried out so far is for given aggregate behavior, focusing on the values of x where the resulting individual behavior is consistent, i.e. coincides, with the aggregate behavior. When this does not happen, it is natural to ask how the aggregate behavior evolves in response to individual behaviors disconfirming it. We posit that the aggregate behavior will decrease over time when $x_i < \overline{x}$, while it will increase when $x_i > \overline{x}$. Avoiding the burden of introducing a formal dynamical model, we rely on this assumption and on the observation from Figure 2 on the shapes of $x_i(\overline{x})$, to conclude that: when only the $x = 1$ solution exists it is an attractor, while, when also another solution exists, this latter solution is attractive and $x = 1$ is no longer so.

2.2 Analytical vs. simulative results

In Figure 3 we observe that there is a discrepancy between analytical and simulative results. In fact, simulations perfectly predict analytical results when cooperation rate under intuition is equal to one and when it is small enough. When cooperation rate under intuition is close to 1, but different from 1, simulations

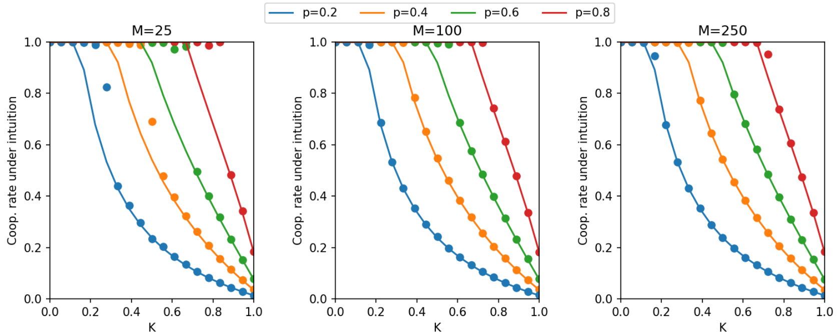

Figure 3: Three different values of M.

tend to overestimate it.

Overestimation occurs with perfect assortativity when, at some point t in time, we have that $R_C^t > R_D^t$ for all agents. In the following periods, all the agents always cooperate under intuition. The system reaches the equilibrium with $x = 1$ and, unless perturbations are introduced, the system can not leave such equilibrium. When two equilibria exist and the the minimum x in those equilibria is close to 1, it is possible that, during the process of convergence to the equilibrium with $x < 1$ (which is the attractor), the dynamic reaches the equilibrium with $x = 1$ due to stochastic realizations of intuitive behavior as cooperation, which is quite likely since x is close to 1. In Figure 3 we show that the greater is the number of agents the lower is the overestimation, because having that all realized behaviors are cooperative becomes less likely.

3 Robustness analysis

3.1 Payoff matrix

The payoff matrices of the two types of interaction are equivalent to the games described by (1), in that they are obtained from theirs by adding c in every entry to avoid negative payoffs.

	C	D
C	b	0
D	$b+c$	c

(a) One shot prisoner dilemma.

	C	D
C	b	c
D	c	c

(b) Repeated prisoner dilemma.

In the simulations presented in the paper we consider $b = 4$ and $c = 1$ as in (1). Results are qualitatively similar if we consider different values of b and c, indeed cooperation rates under intuition monotonically increase as assortativity in cognition increases. Moreover, the lower p, the lower the cooperation rate and also the greater K, the lower the cooperation rate. Furthermore, we notice that the greater is b, maintaining c constant, the greater is the rate of cooperation, see Figure 4.

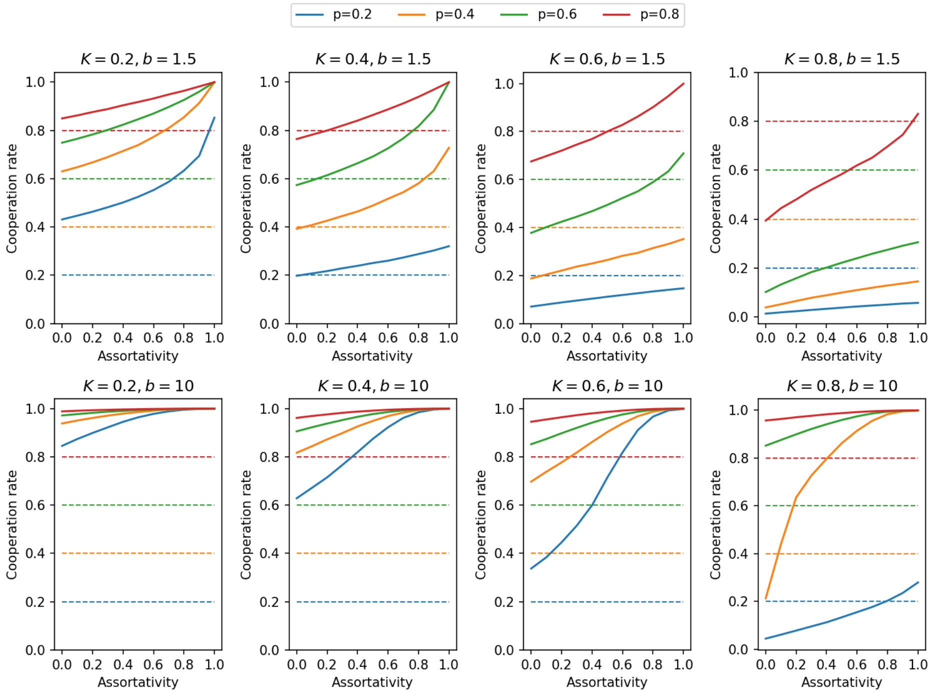

Figure 4: Average cooperation rate varying assortativity in cognition. Each subplot refers to a specific value of K and b. Solid lines represent the average rate of cooperation under intuition, dashed lines represent the average cooperation rate under deliberation, i.e., the value of p. Each color refers to a specific value of p.

3.2 Q-Learning

The standard formulation of Q-learning update is

$$Q_t(x, a) = (1 - \alpha_t)Q_{t-1} + \alpha_t \left[r_t + \gamma V_{t-1}(y_t)\right] \tag{13}$$

where x is the state and a is the action performed, α_t is the learning rate, r_t is the reward obtained in period t, and V_{t-1} is the future value of state y_t that can be reached playing action a in state x, which is discounted by the parameter γ (2). In our formulation, γ is equal to zero, representing the situation in which agents are myopic, i.e., they are unable to make any prediction about future rewards.

Since our results in the main text are given for $\alpha = 0.5$, here we explore their robustness by considering different learning rates. Figure 5 shows the cooperation rate attained under intuition for α taking values 0.25, 0.75 and 1, as K, p, and A change. In particular, K and p range from 0.2 to 0.8 with steps of 0.2, while instead A ranges from 0 to 1 with steps of 0.1. We notice that the quality of results does not vary as α changes, indeed cooperation rates under intuition monotonically increase as assortativity in cognition increases. Moreover, the lower p, the lower the cooperation rate and also the greater K, the lower the cooperation rate. More precisely, we observe that a larger weight given to the past information, i.e., a smaller α, leads to greater cooperation under intuition and, hence, overall.

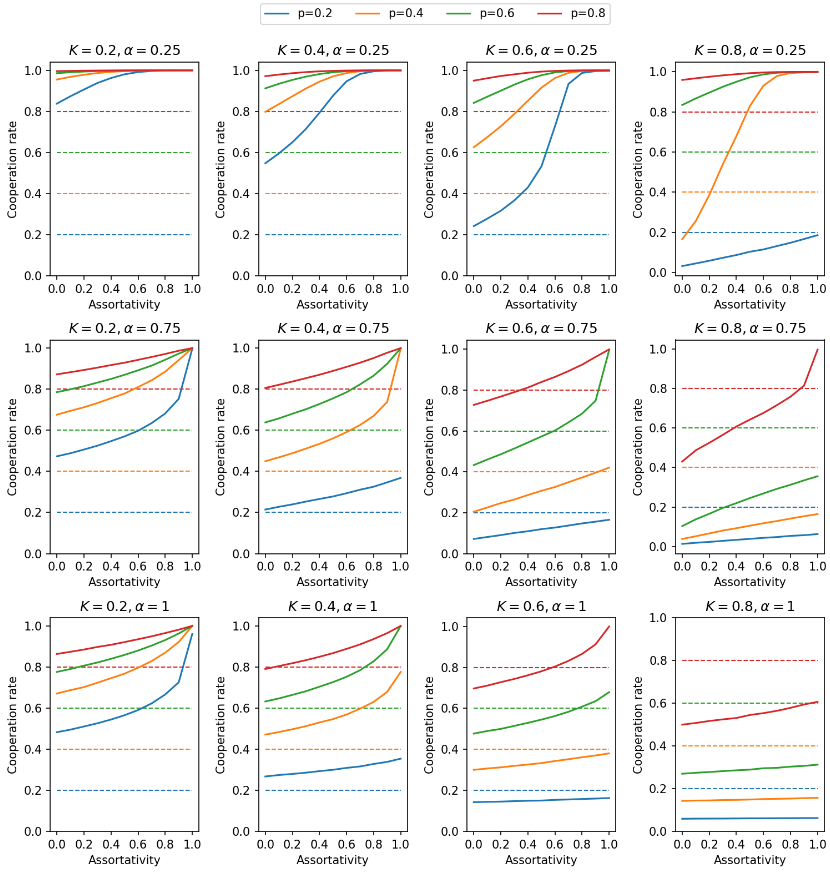

Figure 5: Average cooperation rate for different levels of assortativity in cognition. Each subplot refers to a specific value of K and α. Solid lines represent the average rate of cooperation under intuition, dashed lines represent the average cooperation rate under deliberation (which coincide with p). Each color refers to a specific value of p.

3.3 Q-Learning under deliberation

In this subsection we introduce a learning process for deliberation as well. Agents are characterized by three different memories. The first is the memory through which agents take an intuitive decision, m_i^t, that comprises two elements. One is the information about the past rewards obtained in the previous periods when playing cooperation, $\overline{R}_{i,C}^t$, and the other is information about the past rewards obtained in the previous periods when playing defection, $\overline{R}_{i,D}^t$:

$$m_i^t = \{\overline{R}_{i,C}^t, \overline{R}_{i,D}^t\}$$

The second and the third memories are instead used to take decisions under deliberation, one for each of the two different games. The second memory, $m_{i,0}^t$, comprises two elements: one is $\overline{R}_{i,C,0}^t$, which is a statistics of the payoffs obtained in the past when agent i cooperates under deliberation in the repeated

prisoner dilemma; the other is $\overline{R}_{i,D,0}^t$, which is a statistics of the payoffs obtained in the past when agent i defects under deliberation in the repeated prisoner dilemma. Agent i at time t, when choosing under deliberation in the repeated prisoner dilemma, takes the action associated to the highest between $\overline{R}_{i,D,0}^t$ and $\overline{R}_{i,C,0}^t$. The third memory is $m_{i,1}^t$, and it similarly comprises two elements: $\overline{R}_{i,C,1}^t$ and $\overline{R}_{i,D,1}^t$, which are statistics of past payoffs, for cooperation and defection, respectively, when agent i has deliberated in the one shot prisoner dilemma. Both the memories used for deliberation, $m_{i,0}^t$ and $m_{i,1}^t$, are updated following the same procedure as m_i^t.

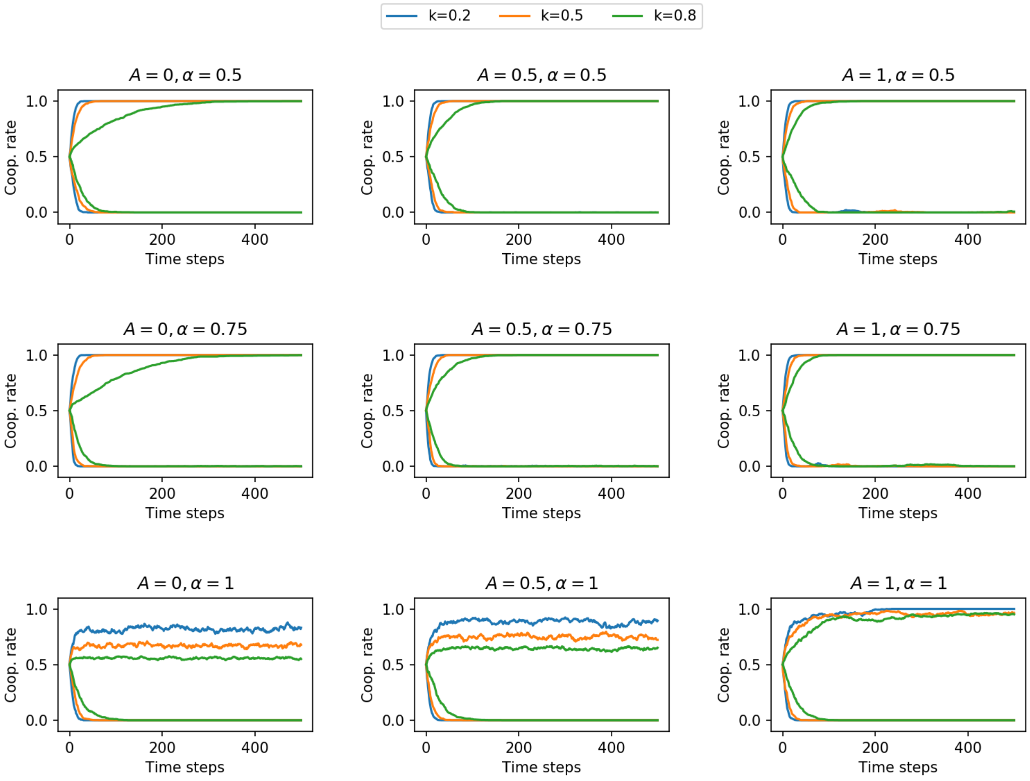

Figure 6: Evolution over time of the average rate of cooperation under deliberation when playing the one shot and the repeated prisoner dilemma for different values of the parameters K, A, and α.

In Figure 6 we plot the evolution in time of the average rate of cooperation under deliberation when playing the one shot and the repeated prisoner dilemma for different values of the parameters K, A, and α. The parameter p is constant and equal to 0.5 to maintain a situation of symmetry between the two games. Colors are related to different values of K, as reported in the legend. In each subplot, for each color, there are two lines, one increasing and the other decreasing. The increasing line is the average cooperation rate under deliberation for the repeated prisoner dilemma, while the decreasing one is the average cooperation rate under deliberation for the one shot prisoner dilemma.

When α is lower than one, i.e., equal to 0.5 and 0.75, agents quickly learn to cooperate under deliberation when they play the repeated prisoner dilemma, while they defect when playing the one shot interaction. In these cases simulations are qualitatively the same of the baseline model: after few iterations, agents cooperate

with probability one when they deliberate in the repeated interaction, while they cooperate with probability zero when they deliberate in the one shot interaction. When $\alpha = 1$, agents are able to learn to defect under deliberation in the one shot prisoner dilemma, while they are not able to completely learn to cooperate in the repeated prisoner dilemma under deliberation. This result is due to the fact that cooperation in the repeated interaction is the weakly dominant strategy, and not the strongly dominant one. Thus, agents who save in memory only the last payoff obtained, given $\alpha = 1$, are often indifferent between the two actions and take their choice randomly.

In general, we observe that the greater is K, the slower is the learning process under deliberation because it is less frequent. Furthermore, the learning process is quicker in the one shot interaction than in the repeated one, which is probably due the different types of dominance (strong as opposed to weak) in the two types of interaction. Moreover, higher assortativity and smaller learning rate speed up the learning process.

4 The optimality of dual process reasoning

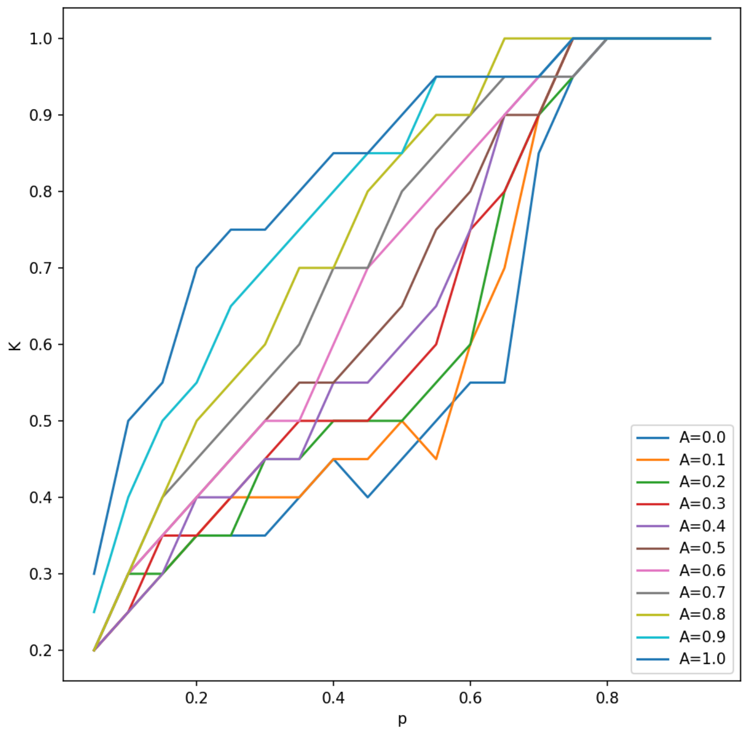

Figure 7: Values of K that maximizes total cooperation varying the parameter p. Different lines with different colors are associated to different values of A.

As we argue in the paper, our aim is not to study the evolution of dual process reasoning. Rather, we assume that agents are dual process reasoners, for which the literature has already provided evolutionary

arguments, and we focus on the effects of assortativity in cognition on cooperation. Nevertheless, it can be interesting to notice that the value of K that, for given p and A, maximizes the overall level of cooperation is often strictly in between 0 and 1. This means that a population of dual process reasoners would perform better than a population of agents that are purely intuitive or deliberative agents.

A number of remarks can be done by looking at Figure 7. First, we notice that the value of K that maximizes total cooperation increases as p increases. In other words, a higher probability of repeated interactions, for which cooperation performs better than defection at the individual level, makes deliberation less important to maximize cooperation. Second, the higher the assortativity in cognition, the greater is the optimal value of K. Indeed, when assortativity is high, deliberation is more effective in shaping the heuristics, and thus less deliberation is needed to sustain cooperation. Third, we notice that populations of purely deliberative agents (i.e., $K = 0$) are never optimal, while populations of purely intuitive agents (i.e., $K = 1$) can be optimal, which happens when p is high enough. These remarks appear not be fully general by looking at the figure (see, for instance, that the lines are not always monotonically increasing), but this can be an artifact of a rather limited number of simulations, also considering that we find a tiny difference of cooperation rates between the maximizing K and the level of K attaining the second-highest cooperation rate.

References

[1] Adam Bear and David G Rand. Intuition, deliberation, and the evolution of cooperation. *Proceedings of the National Academy of Sciences*, 113(4):936–941, 2016.

[2] Christopher JCH Watkins and Peter Dayan. Q-learning. *Machine Learning*, 8(3):279–292, 1992.
